# Significance of Medicinal Mushrooms in Integrative Oncology: A Narrative Review

**DOI:** 10.3389/fphar.2020.580656

**Published:** 2020-11-11

**Authors:** Michael Jeitler, Andreas Michalsen, Daniela Frings, Marisa Hübner, Moritz Fischer, Daniela A. Koppold-Liebscher, Vijay Murthy, Christian S. Kessler

**Affiliations:** ^1^ Institute for Social Medicine, Epidemiology and Health Economics, Charité – Universitätsmedizin Berlin, corporate member of Freie Universität Berlin, Humboldt-Universität zu Berlin, and Berlin Institute of Health, Berlin, Germany; ^2^ Department of Internal and Integrative Medicine, Immanuel Hospital Berlin, Berlin, Germany; ^3^ Australian Research Centre in Complementary and Integrative Medicine, University of Technology Sydney, Sydney, NSW, Australia

**Keywords:** medicinal mushrooms, integrative oncology, integrative medicine, medicinal fungi, complementary medicine, Integrative medicine

## Abstract

Medicinal mushrooms are widely used in East Asia for the treatment of various diseases, especially in complementary cancer care. While there is a growing interest in medicinal mushrooms in Western countries and an increasing number of pre-clinical studies indicate distinct anti-cancer and regenerative properties, little is known about their potential relevance for clinical practice. This review aims to provide an overview of the clinical evidence, significance and potential role of medicinal mushrooms in complementary cancer care. Scientific databases for (randomized) controlled clinical trials evaluating whole spectrum formulations of medicinal mushrooms (mushroom powder and mushroom extracts) in cancer patients during and/or after conventional oncological treatment were searched. Eight studies met our inclusion criteria (eight randomized controlled trials, one controlled clinical trial). The medicinal mushrooms investigated were *Agaricus sylvaticus* (two trials), *Agaricus blazei murill* (two trials), *Antrodia cinnamomea* (one trial), *Coriolus versicolor* (one trial) and *Ganoderma lucidum* (three trials); all were compared to placebo and administered orally. A variety of cancer entities, outcomes and treatment durations were observed. Study results suggested beneficial effects of medicinal mushrooms, particularly quality of life and reduction of adverse effects of conventional therapies. Also, positive effects on antitumor activity and immunomodulation were reported, e.g., an increased activity of natural killer cells. In addition, results might suggest a longer survival of cancer patients receiving mushroom preparations, although in most studies this was not significant when compared to placebo. Adverse events of treatment with medicinal mushrooms were poorly reported; gastrointestinal reactions and a decrease in platelet cell count occurred in some cases. The methodological quality of most studies was generally unsatisfying and most results were insufficiently reported in several respects. Medicinal mushrooms may have a therapeutic potential for cancer patients during and after conventional oncological care with regards to quality of life, reduction of adverse effects of conventional care and possibly other surrogate parameters like immune function. There is an urgent need to investigate the safety and possible interactions of medicinal mushrooms. High-quality clinical research is warranted in order to clarify the potential of medicinal mushrooms in cancer therapy.

## Introduction

Cancer is a major global threat and is the second leading cause of death in industrialized countries after cardiovascular diseases (Global Burden of Disease Cancer Collaboration et al., 2017). Conventional cancer treatments include surgery, chemotherapy, radiation, hormone-targeted therapies and immunotherapies. These treatments can have various side effects such as gastrointestinal symptoms, skin or mucous membrane irritation, pain, fatigue, bone marrow suppression or immunosuppression, which may lead to higher infection rates and can significantly affect quality of life during or post oncological treatments (Urruticoechea et al., 2010).

Despite the growing success of conventional personalized cancer therapies, recurrence and metastases remain common, depending on the type of cancer and the stage of disease (Miller et al., 2019). In addition, a large number of patients are diagnosed when in advanced stages, leaving little treatment options but for palliative care (Haun et al., 2017).

The use of complementary and integrative medicine (CIM) is widespread and widely accepted in cancer patients (Astin, 1998; Ernst and Cassileth, 1998). Studies have shown that 20–50% of cancer patients use some form of CIM therapies as an add-on to conventional treatments (Ernst and Cassileth, 1998; Grant et al., 2019). Medicinal mushrooms have been used in traditional Asian medical systems continually for several centuries (Wasser, 2010).

Mushrooms have been identified to possess many medicinal functions (Wasser, 2010; Wasser, 2014), including antioxidant (Geng et al., 2016), antimicrobial, anti-inflammatory (Shigesue et al., 2000), immunomodulating (Gao et al., 2013), antitumoral (Fan et al., 2011; Reis et al., 2015), hepatoprotective (Zhang et al., 2002), antidiabetic (Kim et al., 2010), prebiotic properties (Singdevsachan et al., 2016). These properties were primarily demonstrated in *in vitro* studies. Mushrooms are high in protein (up to 40%), vitamins, fiber, minerals, trace elements and are low in calories and cholesterol (Wasser, 2010). Higher basidiomycetes mushrooms (pillar mushrooms/stand mushrooms) contain various types of biologically active, high-molecular (e.g., ß-glucans) and low-molecular compounds (e.g., triterpenes, lectins, steroids) in fruiting bodies, spores, culture mycelia and culture broth, with suggested anticarcinogenic effects (Wasser and Weis, 1999; Wasser, 2017). They contain various compounds that intervene in signaling pathways of tumor-specific proliferation, regulation of apoptosis, cancer-specific metabolism, angiogenesis, metastasis and key functions of the immune system (Blagodatski et al., 2018).

Several extracts from medicinal mushrooms such as polysaccharides and in particular ß-glucans, e.g., krestin and polysaccharide peptide (PSP) from *Coriolus versicolor*, and lentinan, isolated from *Lentinula edodes* (Shiitake), have been investigated *in vivo*, *in vitro* and in studies in humans (Joseph et al., 2018). Many clinical studies have been conducted on the complementary use of these substances, e.g., in conjunction with chemotherapy. The results indicate relevant health benefits such as overall disease-free survival of colorectal cancer patients and in improving quality of life among lung cancer patients (Sakamoto et al., 2006; Zhang et al., 2018). A wide range of antitumoral or immunostimulating polysaccharides with different chemical structure from higher basidiomycetes mushrooms has been widely investigated (Wasser, 2002). Medicinal mushrooms have been approved for more than 30 years in Japan and China as an adjunct to standard cancer treatments and have an extensive clinical history of safe use as a single agent or in combination with chemotherapy (Rossi et al., 2018).

Although very popular in East Asia, medicinal mushroom therapies are still largely unknown in Western countries, especially in conventional medical institutions. The medicinal use of mushrooms is becoming wide known within the CIM communities of Europe and the United States. The number of studies on medical mushrooms in oncology has increased in recent decades (Joseph et al., 2018). Apart from focusing on certain mushroom-derived mono-substances, such as PSP, lentinan and krestin, research into the entire spectrum of mushroom formulations is considered promising. The large number of active ingredients of medicinal mushrooms is suspected to synergistically act on several cancer-related pathways (Wasser, 2017; Joseph et al., 2018).

A single mushroom species may contain several of active ingredients which have the potential to influence several cancer-related processes in a synergistic way (Blagodatski et al., 2018; Joseph et al., 2018). As such research on complex synergistic anticancer effects caused by combinations of molecules in mushroom extracts seems particularly important.

The purpose of this narrative review was to evaluate clinical trials (CTs) investigating the effects of medicinal mushrooms in the supportive, complementary therapy of cancer patients during and/or post conventional care. They are currently mostly multi-substance formulations and are available in the form of powders, tablets, capsules or other extract forms. In this paper we focused on clinical studies which investigated the effects of these formulations on immune function, quality of life, well-being and their adverse effects.

## Methods

We searched MEDLINE, EMBASE CENTRAL, the Web of Science, ClinicalTrials.gov and the WHO International CTs Registry Platform Search Portal from inception until June 2020, with no restrictions on the date of publication. Studies were included, if they 1) investigated cancer patients, 2) included whole spectrum mushroom formulations, 3) were in English language, and 4) were used as complementary therapy during or after conventional cancer treatment. Studies were excluded, if they 1) had study designs other than controlled CT or randomized controlled trials (RCT), 2) were animal studies or *in vitro* studies, or 3) investigated participants with no cancer diagnosis. The study protocol was reviewed and approved by the ethics committee of the Charité-University Medical Center, Berlin, Germany, registration number EA1/172/19. The study was not registered at a CTs registry.

## Results

Our search yielded a total of 272 studies from the electronic databases. After sorting out repetitive hits, irrelevant and several single arm studies with various outcomes, e.g., dose escalation studies (Kodama et al., 2002; Gao et al., 2003b; Yoshimura et al., 2010; Palomares et al., 2011; Torkelson et al., 2012; Ohno et al., 2013; Suzuki et al., 2013; Twardowski et al., 2015) as well as many publications on *in vitro* and animal models, nine studies met our inclusion and exclusion criteria for a narrative review on medicinal mushrooms in human cancer therapy (eight RCTs, one CCT) (Table 1). A large variety of cancer entities, studied sample sizes, outcomes, treatment durations and observation times were seen (Table 1). The medicinal mushrooms investigated were *Agaricus sylvaticus* (two trials) (Fortes et al., 2008; Costa Fortes et al., 2010; Costa Fortes and Carvalho Garbi Novaes, 2011; Valadares et al., 2013), *Agaricus blazei murill* (two trials) (Ahn et al., 2004; Tangen et al., 2015), *Antrodia cinnamomea* (one trial) (Tsai et al., 2016), *Coriolus versicolor* (one trial) (Chay et al., 2017) and *Ganoderma lucidum* (three trials) (Gao et al., 2003a; Oka et al., 2010; Zhao et al., 2012). All mushrooms were administered orally. Four articles were by Fortes et al. and they described different results of a study with *Agaricus sylvaticus* which included 56 colorectal cancer patients (Fortes et al., 2008; Fortes et al., 2009; Costa Fortes et al., 2010; Costa Fortes and Carvalho Garbi Novaes, 2011). These four articles were presented as one study. Most studies were named as placebo-controlled, double-blind RCTs. The methodological quality of most studies was generally unsatisfactory and most results were poorly reported in many aspects.

**TABLE 1:** Overview of studies included in this review.

Reference	Type of Cancer	Oral application	Species Name	Type of Study	Number of Patients	Duration of Treatment	Parameters	Main results
(Valadares et al., 2013)	Breast cancer, stage II and III receiving chemotherapy	Extract (2.1 g/day), divided into 2 daily doses (6 tablets per day, 3 in the morning and 3 in the afternoon, between meals) for a period of 6 months	*Agaricus* sylvaticus	RCT, placebo-controlled, double-blind	46	6 months	Chemotherapy-associated adverse effects	Patients in the verum group had reduced chemotherapy-associated adverse effects (nausea, vomiting and anorexia)
(Fortes et al., 2008; Fortes et al., 2009; Costa Fortes et al., 2010; Costa Fortes and Carvalho Garbi Novaes, 2011)	Postsurgical patients with colorectal cancer stages I-III	Extract (30 mg/kg/day), divided into 2 daily doses (6 tablets per day, 3 in the morning and 3 in the afternoon, between meals)	*Agaricus* sylvaticus	RCT, placebo-controlled, double-blind	56	6 months	Blood count, iron, fasting glucose, BMI, blood pressure	Beneficial effects in the verum group regarding hematological parameters and a reduction of fasting plasma glucose levels and blood pressure. No effects on BMI.
(Tangen et al., 2015)	Multiple myeloma undergoing high dose chemotherapy and autologous stem cell transplantation	Extract, 60 ml/day	AndoSan (82.4% of *Agaricus blazei* Murill, 14.7% of hericium erinaceus, and 2.9% of grifola frondosa)	RCT, placebo-controlled, double-blind	40	Intake 7 weeks	Primary end points: 1) changes in serum levels of cytokines, chemokines, and growth factors in peripheral blood, 2) differences in expression levels of genes involved in immune activation by whole genome assay, both measured on the day of inclusion and at the end of intake of the study product, and 3) differences in the stem cell harvest product of a number of mononuclear cell subsets associated with the immune system. Secondary end points: 1) clinical response to treatment, 2) time in neutropenia, 3) days with body temperature above 38°C, 4) days with IV antibiotics after stem cell infusion, 5) time to new treatment, 6) OS, and 7) quality of life	In the leukapheresis product harvested after stem cell mobilization, increased percentages of treg cells (CD4^+^/CD127d^+^/CD25) and plasmacytoid dendritic cells (CD303^+^) were found in patients receiving verum. A significant increase in serum levels of IL-1, IL-5 and IL- 7 was observed at the end of treatment in the verum group.Increased expression of immunoglobulin genes, killer immunoglobulin receptor genes and HLA genes were observed in the verum group there were no statistically significant differences in treatment response, OS, time in neutropenia, temperature above 38 °C and time to new treatment in the verum group, trends were observed for a longer median time to next treatment and a shorter duration of IV antibiotic treatment. Health related quality of life assessment revealed no differences between the study groups
(Ahn et al., 2004)	Cervical, ovarian, endometrial cancer undergoing chemotherapy	Extract, 3 packs/day, 1 pack per time	*Agaricus blazei* Murill Kyowa	RCT, placebo-controlled, double-blind	100	Every 3 weeks for at least 3 cycles of chemotherapy	Immunological status, side effects, quality of life	Natural killer cell activity was significantly higher in the verum group. No significant difference in lymphokine-activated killer and monocyte activities was observed in a manner similar to the count of specific immune cell populations between the groups. Chemotherapy-associated side effects such as appetite, alopecia, emotional stability, and general weakness were improved by verum only
(Tsai et al., 2016)	Advanced and/or metastatic adenocarcinoma (breast, gastric, lung, liver, colorectal), previously treated with standard chemotherapy	Extract, 20 ml, 2×/day	Antrodia cinnamomea	RCT, placebo-controlled, double-blind	37	30 days	The primary endpoint was 6-months OS; the secondary endpoints were DCR, QoL and biochemical parameters	Mean OSs were 5.4 months for the verum group and 5.0 months for the placebo group (*p* = 0.340), and the DCRs were 41.2 and 55%, respectively (*p* = 0.33). Most hematologic, liver, or kidney functions did not differ significantly between the two groups, but platelet counts were lower in the verum group than in the placebo group (*p* = 0.02). QoL assessments were similar in the two groups, except that the verum group showed significant improvements in quality of sleep (*p* = 0.04)
(Chay et al., 2017)	Advanced hepatocellular carcinoma	Extract, 2.4 g/day	Coriolus versicolor	RCT - 2:1 to either verum or placebo	15	Median treatment time 2.5–4.2 months	The primary endpoint was the median TTP between both arms. Secondary endpoints include evaluating response rates, toxity, qol, PFS and OS.	There was no difference in TTP with use of verum compared to placebo. Verum had better social and emotional functioning scores and less appetite loss and pain symptoms compared to placebo subjects during treatment
(Zhao et al., 2012)	Breast cancer patients with cancer-related fatigue undergoing endocrine therapy	Spore powder, 1 g 3×/day for 4 weeks	*Ganoderma lucidum*	RCT, placebo-controlled	48	4 weeks	FACT-F, HADS, EORTC QLQ-C30, TNF-α, IL-6	The verum group showed statistically significant improvements in the domains of physical well-being, fatigue and overall quality of life and less anxiety/depression after intervention compared to placebo. TNF-*α* and IL-6 were lower in the verum group compared to placebo
(Oka et al., 2010)	Colorectal adenoma	Extract, 1.5 g/day	*Ganoderma lucidum*	CCT	198	12 months	Size, site and macroscopic type of all adenomas	The changes in the number of adenomas up to 12 months increased to 0.66 ± 0.10 in the control group, while decreasing in the verum group to −0.42 ± 0.10 (*p* < 0.01). The total size of adenomas increased to 1.73 ± 0.28 mm in the control group and decreased to −1.40 ± 0.64 mm in the verum group (*p* < 0.01)
(Gao et al., 2003a)	Advanced lung cancer	Extract, 600 mg 3×/day	*Ganoderma lucidum*	RCT, placebo-controlled, double-blind	68	12 weeks	Karnofsky performance status, immunological parameters	Treatment with verum showed a stable disease in 35% lung cancer patients at the 12-weeks visit, which was significantly higher than in the control group (22%). Treatment with verum resulted in a significant increase in karnofsky scores (>10 scores) in 50% of the patients compared to placebo (14% in placebo group) cancer-related symptoms (e.g., fever, cough, weakness, sweating and insomnia) were significantly improved in 43–84% of cancer patients receiving verum compared to placebo (11–43%). Verum showed a significant increase in lymphocyte mitogenic reactivity to concanavalin A, CD3 percentage and natural killer cell activity compared to placebo

RCT, Randomized Controlled Trial; CCT, Controlled Clinical Trial; BMI, Body Mass Index; OS, overall survival; DCR, disease control rate; QoL, quality of life; TTP, time to progression; PFS, progression-free survival; EORTC-QLQC30, European organization for research and treatment - quality of life questionnaire; HADS, Hospital Anxiety and Depression Scale; FACT-Hep, Functional Assessment of Cancer Therapy - Hepatobiliary Cancer; IV, intravenous; CD, cluster of differentiation; IL, interleukin; TNF, tumor necrosis factor

According to the included studies medicinal mushrooms appear to facilitate improvements in: 1) quality of life, 2) a reduction of side effects by conventional therapy (e.g., chemotherapy), 3) hematologic parameters, 4) overall survival (OS), antitumor activity and immunomodulation. Furthermore 5) medicinal mushrooms seem to be safe. These points are presented in detail below:

### Quality of Life

Chay et al. investigated effects of *Coriolus versicolor* as a complementary therapy in a RCT with 15 patients with advanced hepatocellular carcinoma (HCC) who had poor liver function or were ineligible for standard therapy (Chay et al., 2017). Participants in the verum group reported improved emotional, physical, social and cognitive function compared to the placebo group on the European organization for research and treatment - quality of life questionnaire (EORTC-QLQC30) during the treatment. However, this was not statistically significant between the groups. Based on the Functional Assessment of Cancer Therapy - Hepatobiliary Cancer (FACT-Hep) questionnaire, the lowest values for physical and emotional well-being reported by the subjects in the verum group were on average higher than those reported by patients receiving placebo during the treatment.

Fortes et al. investigated 56 patients with colorectal cancer after surgery over a period of 6 months, randomly assigned to the intake *Agaricus sylvaticus* or placebo (Costa Fortes et al., 2010). Analyses did not show significant differences regarding quality of life, however the results after 6 months showed a tendency toward better mood and sleep, and less gastrointestinal symptoms and pain in the verum group.

Tsai et al. observed quality of life in a RCT (Tsai et al., 2016). Thirty seven patients with advanced adenocarcinomas of breast, lungs, stomach, liver and colorectal region undergoing chemotherapy were enrolled and randomized to a 30-days-supplementary treatment with *Antrodia cinnamomea* or placebo. Assessments of the EORTC-QLQ-C30 showed no significant differences between the groups other than sleep, which was significantly improved in the verum group (*p* = 0.04).

Ahn et al. conducted a RCT including 100 patients with different gynecological cancers (cervical, endometrium and ovarian) receiving chemotherapy and *Agaricus blazeii murill* Kyowa or placebo (Ahn et al., 2004). Patients showed improvements in terms of mood parameters (anxiety, depression, mental stability) and body strength compared to placebo on a modified EORTC-QLQ-C30. Between-group differences were not reported.

In a RCT of Zhao et al. investigating 48 breast cancer patients with cancer-related fatigue undergoing endocrine therapy the subscales on the EORTC QLQ-C30 physical function (verum: week 0: 63.7 ± 25.9, week 4: 78.2 ± 26.1; placebo: week 0: 64.0 ± 27.1, week 4: 64.5 ± 28.7) and global quality of life (verum: week 0: 55.8 ± 22.9, week 4: 68.9 ± 21.4; placebo: week 0: 56.6 ± 23.0, week 4: 57.7 ± 24.2) were significantly (*p* < 0.01) improved after a 4-weeks treatment with spore powder of *Ganoderma lucidum* compared to placebo (Zhao et al., 2012). Also, the parameters fatigue (verum: week 0: 39.76 ± 5.10, week 4: 46.78 ± 5.07; placebo: week 0: 40.35 ± 6.10, week 4: 40.92 ± 5.62), sleep disturbance (verum: week 0: 56.5 ± 21.8, week 4: 42.3 ± 26.2; placebo: week 0: 55.8 ± 22.6, week 4: 53.9 ± 24.8) and loss of appetite (verum: week 0: 32.5 ± 19.3, week 4: 24.3 ± 18.4; placebo: week 0: 32.3 ± 17.4, week 4: 30.3 ± 16.5) among patients in the verum group improved significantly (*p* < 0.01, *p* < 0.01 and *p* < 0.05, respectively) on the EORTC QLQ-C30 and FACT-F. In addition, anxiety (verum: week 0: 6.3 ± 3.2, week 4: 4.1 ± 2.9; placebo: week 0: 6.5 ± 3.4, week 4: 6.1 ± 3.2) and depression (verum: week 0: 4.9 ± 3.8, week 4: 3.1 ± 2.8; placebo: week 0: 4.8 ± 3.1, week 4: 4.6 ± 2.9) on the Hospital Anxiety and Depression Scale improved significantly (*p* < 0.05 and *p* < 0.01, respectively) compared to the control group.

Health related quality of life assessment revealed no differences between the study groups on the EORTC QLQ-C 30 questionnaire in a RCT with patients with multiple myeloma undergoing high dose chemotherapy and autologous stem cell transplantation (verum: *Agaricus blazei Murill*) (Tangen et al., 2015).

In a RCT of Gao with patients with advanced lung cancer treatment with *Ganoderma lucidum* resulted in a significant increase in Karnofsky scores (i.e., in >10 scores) in 50% of the 32 verum patients compared to placebo (in comparison to 14% of the 29 patients in the placebo group) (Gao et al., 2003a). Nine (28%) and seven (22%) patients receiving verum had unchanged and reduced Karnofsky scores in comparison to 13 (46%) and 11 (39%) in the placebo group.

### Reduction of Side Effects by Conventional Therapy

Valadares et al. focused on the effects of *Agaricus sylvaticus* extract (2.1 g/day for a period of 6 months) on side effects of chemotherapy in breast cancer patients in a RCT (Valadares et al., 2013). Patients with three cycles of chemotherapy at the beginning of treatment reported poor appetite, with 23% of these patients in the placebo group and 54% of these patients in the *Agaricus sylvaticus* group. After 3 months of treatment with *Agaricus sylvaticus*, 31% of patients in the placebo group reported a reduction in appetite, while no such symptoms occurred in the supplemented group. Compared to patients with six cycles of chemotherapy and use of *Agaricus sylvaticus*, the data showed that, toward the end of the treatment, loss of appetite decreased over time. In comparison, 80% of patients in the placebo group complained about loss of appetite by the end of the study after 6 months. While the majority of the placebo-receiving patients suffered from gastrointestinal symptoms like diarrhea, constipation, nausea and vomiting, only two patients in the verum group reported these side effects. Patients taking medicinal mushrooms had nearly no loss of appetite and only in a few cases fever was an occurrence while this was also frequent in the placebo group.

In the RCT of Chay et al. lower average symptom scores for nausea, vomiting, pain, insomnia, constipation, and diarrhea were reported by patients supplemented with *Coriolus versicolor* during conventional treatment of HCC patients compared to placebo (Chay et al., 2017). Verum patients also experienced significantly less pain (group difference: −38.6 95% CI: 65.5 to −11.8; *p* = 0.011) and loss of appetite (group difference: 39.7 95% CI: 64.5 to −15.0, *p* = 0.006) during treatment compared to placebo patients.

Ahn et al. showed in a RCT that patients with gynecological cancers receiving chemotherapy suffered from fewer side effects such as loss of appetite, alopecia, emotional instability and general weakness when the therapy was complemented with *Agaricus blazeii murill* compared to placebo (Ahn et al., 2004). Participants completed a questionnaire that sought data on physical and emotional status of the patients. These include insomnia, appetite, alopecia, body weight, nausea/vomiting, emotional conditions, discomfort, and general body strength. In this study a figure only reported these results. Detailed results were not available.

In the RCT of Tsai et al. patients with advanced adenocarcinomas were treated with *Antrodia cinnamomea* alongside chemotherapy. While gastrointestinal symptoms such as abdominal pain and diarrhea were observed more frequently in the verum than in the placebo group (8 vs. 6)., their intensity was lower (grade 1 and 2) compared to the placebo group (grade 3 and 4) (Tsai et al., 2016). Four patients suffered severe abdominal pain receiving placebo due to disease progression.

In advanced lung cancer, several cancer-related symptoms such as fever, cough, weakness, sweating and insomnia improved significantly in 43–84% of patients receiving *Ganoderma lucidum* compared to placebo (11–43%) (Gao et al., 2003a).

### Hematological Parameters

Fortes et al. observed in a RCT beneficial hematological and glycaemic effects of dietary supplementation with *Agaricus sylvaticus* in patients with colorectal cancer after surgery (Fortes et al., 2008; Costa Fortes et al., 2010; Costa Fortes and Carvalho Garbi Novaes, 2011). The verum group showed significant within-group reductions of fasting plasma glucose, cholesterol, creatinine, aspartate aminotransferase, alanine aminotransferase, immunoglobulin A, immunoglobulin M, systolic and diastolic blood pressure and an improvement in hematological parameters (e.g., hemoglobin, hematocrit, erythrocytes, iron) after 3 and 6 months of treatment, whereas in the placebo group no significant within-group changes were observed. No effects on weight and body mass index were observed. Between-group differences were not calculated.

In the RCT of Tsai et al. most hematological, liver or kidney functions did not differ significantly between the two groups, but platelet counts were lower in the *Antrodia cinnamomea* group than in the placebo group (*p* = 0.02) (Tsai et al., 2016).

### Survival, Antitumor Activity and Immunomodulation

In the latter RCT the supplemental therapy with *Antrodia cinnamomea* for chemotherapy in advanced adenocarcinomas showed no significant difference in OS compared to placebo (Tsai et al., 2016). Progression of the disease was the leading cause of death in four (33.3%) verum and eight (66.7%) placebo recipients. Mean OS was 5.4 months in the verum group and 5 months in the placebo group (*p* = 0.340), and disease control rates were 41.2% and 55% respectively (*p* = 0.33).

In the RCT with patients with advanced HCC treated with *Coriolus versicolor*, trends toward longer median OS compared to placebo (6.5 vs. 2.2 months, *Coriolus versicolor* vs. placebo) and median progression-free survival (2.5 vs. 1.1 months) were observed (Chay et al., 2017). The time to progression (TTP) was not significant (2.5 vs. 4.2 months).

Oka et al. showed in a CT that a water-soluble extract from the culture medium of *Ganoderma lucidum* mycelia suppressed the development of colorectal adenoma (Oka et al., 2010). Follow-up colonoscopy was performed after 12 months, 96 patients completed the trial. One hundred two patients in the no-treatment control group were selected randomly. The changes in the number of adenomas up to 12 months increased to 0.66 ± 0.10 in the control group, while decreasing in the verum group to −0.42 ± 0.10 (*p* < 0.01). The total size of adenomas increased to 1.73 ± 0.28 mm in the control group and decreased to −1.40 ± 0.64 mm in the verum group (*p* < 0.01).

Chay et al. observed no difference in the use of *Coriolus versicolor* compared to placebo regarding the primary endpoint TTP in advanced HCC treated with chemotherapy. The median duration of the treatment in the placebo and verum arm was 1.5 cycles and three cycles, respectively. Median TTP was 2.5 (1.4–5.3) months compared to 4.2 (0.4–4.2) months in the verum and placebo arm, respectively, with a hazard ratio (HR) of 0.70 (0.16–3.05 *p* = 0.634). Median progression-free survival was 2.5 (1.4–5.3) months in the verum and 1.1 (0.4–4.2) months in the placebo arm, HR 0.42 (0.13–1.34, *p* = 0.144). Median OS was 6.5 (3.3–24.1) and 2.2 (0.8–23.3) months, respectively, HR 0.35 (0.10–1.25, *p* = 0.105). In addition, the group examined the serum concentration of potentially relevant markers for HCC. They found a decrease in interleukin (IL)17F, MCP-1 and increase in prolactin and TNF-related apoptosis-inducing ligands in patients with HCC treated with *Coriolus versicolor* compared to placebo (Chay et al., 2017).

The activity of natural killer cells in patients with gynecological cancers undergoing chemotherapy was significantly increased in the verum group treated with *Agaricus blazei murill* after 3 and 6 weeks compared to placebo (Ahn et al., 2004). No difference was observed in the number of white blood cells, monocytes, lymphocytes, T cells, cluster of differentiation (CD) 48^+^ and CD 56^+^ cells.

In an RCT Tangen et al. investigated the effects of *Agaricus blazei Murill* supplementation on multiple myeloma patients undergoing high dose chemotherapy and autologous stem cell transplantation. They found no statistically significant differences in treatment response, OS and time of treatment between patients supplemented with AndoSan (containing 82.4% of *Agaricus blazei Murill*) and placebo (Tangen et al., 2015). In the verum group, trends toward a longer median time to the next treatment (37.5 vs. 31.2 months) and a shorter duration of intravenously (IV) antibiotic treatment (8.6 vs. 10.0 days) were observed. Moreover beneficial effects on immunological parameters were found in the verum group only: 1) a significant increase in serum levels of IL 1ra, IL-5 and IL-7, 2) an increased expression of immunoglobulin genes, KIR genes and HLA-genes and 3) in the leukapheresis product harvested after stem cell mobilization, increased percentages of Treg cells (CD4^+^/CD127d^+^/Cd25^+^) and plasmacytoid dendritic cells (CD303^+^).

*Ganoderma lucidum* supplementation resulted in a stable disease condition in 35.1% of the study population suffering from lung cancer at the 12 week’s visit (Gao et al., 2003a). This was significantly higher than in the control group (22.6%). Moreover, a significant increase in lymphocyte mitogenic reactivity to concanavalin A (baseline: 52.3 ± 11.5, after treatment: 68.9 ± 8.9, *p* < 0.05), CD3 percentage (baseline: 46.2 ± 11.3, after treatment: 55.8 ± 10.5, *p* < 0.05) and natural killer cell activity (baseline: 24.1 ± 12.3, after treatment: 42.8 ± 19.7, *p* < 0.05) was shown; a marginal increase in CD4 percentage and CD4/CD8 ratio; but a marginal decrease in CD8. All of these immune parameters remained unchanged or decreased in the control group.

### Safety of Medicinal Mushrooms

Adverse events of treatment with medicinal mushrooms were poorly reported. Only in three trials there was an explicit reference to such events (Oka et al., 2010; Zhao et al., 2012; Tsai et al., 2016). No serious adverse events due to medical mushroom intake were reported.

Adverse effects attributed to the mushrooms were a significant decrease in platelet cell count within normal ranges during a 30-days treatment with *Antrodia cinnamomea* and a 6-months treatment with *Agaricus sylvaticus* (Fortes et al., 2009; Tsai et al., 2016), while all other laboratory values in the verum groups were stable or improved.

In the CCT among patients with colorectal adenoma treated with *Ganoderma lucidum* adverse events were noted in six out of 123 patients in the verum group (Oka et al., 2010). Symptoms were diarrhea (n = 4), stomach discomfort (n = 1) and poor health (n = 1). Treatment with the verum was discontinued in all cases. No adverse events were reported for the placebo group (n = 102).

The incidence of gastrointestinal reactions, including abdominal pain and diarrhea, was much greater in the *Antrodia cinnamomea* group (47%) than in the placebo group. The adverse events observed in patients treated with *Antrodia cinnamomea* were generally consistent with its known adverse event profile (Tsai et al., 2016).

In the RCT of Zhao et al. examining breast cancer patients with cancer-related fatigue undergoing endocrine therapy treated with *Ganoderma lucidum* no serious adverse effects were reported during the study. Only “mild discomfort” was recorded, and these are shown in a table for the verum group only. The two most common discomfort were dizziness (16%) and dry mouth (12%) (Zhao et al., 2012).

The study using *Ganoderma lucidum* in advanced lung cancer describes three episodes of mild toxicity (nausea: 2; insomnia: 1) in the verum and one episode of toxicity (vomiting) in the control group (Gao et al., 2003a).

## Discussion

The studies evaluated in this narrative review suggest that the use of medicinal mushrooms may improve cancer-related and treatment-related symptoms in patients with different types and stages of cancer when used as a complementary therapy alongside conventional cancer care. Most studies considered in this paper showed beneficial effects in favor of medicinal mushrooms for various cancer entities, especially with regards to quality of life and reduction of side effects of conventional oncological treatment (particularly chemotherapy and endocrine therapy). Also, positive effects on antitumor activity and immunomodulation were reported, e.g., an increased activity of natural killer cells. In addition, there was some uncertain evidence of a longer survival of cancer patients treated with medicinal mushrooms, although in most studies the effect was not significant when compared to placebo. Adverse or negative effects of the used extracts were rarely reported. The only reported adverse effects being gastrointestinal reactions and decrease in platelet count. No serious adverse event due to medical mushroom intake was reported in the reviewed studies.

Reduced quality of life and fatigue are common side effects of conventional cancer treatment and the disease itself (Visser and Smets, 1998; Bottomley, 2002). Most studies observed improvements in various aspects of quality of life. Moreover, emotional and mental well-being, and mood scores (particularly anxiety and depression) also improved in some studies (Ahn et al., 2004). A Cochrane review of *Ganoderma lucidum* for cancer treatment showed that patients in the *Ganoderma lucidum* group had a relatively better quality of life compared to controls (Jin et al., 2016). Moreover, sleep disorders affect 30–50% of cancer patients and contribute to additional risks of depression, fatigue, increased pain and reduced survival rates (Otte et al., 2015). Some studies in this review showed an increased quality of sleep associated with medicinal mushroom intake (Fortes et al., 2008; Tsai et al., 2016; Chay et al., 2017). In addition, several mushroom species are suspected to have an anti-fatigue effect (Geng et al., 2017).

Also, reduction of chemotherapy-related adverse effects was reported. Several common side effects (nausea, vomiting, pain and hair loss) were reduced in most of the studies reviewed in this paper.

Leukopenia, especially lymphopenia and neutropenia, are consequences of cachexia and the metabolic changes caused by the tumor and increase the risk of infection (Wasser and Weis, 1999). In patients with multiple myeloma, the immune status was much better in terms of white blood cells and immunoglobins, which also led to fewer infections when treated with *Agaricus blazei murill* (Tangen et al., 2015). Medicinal mushrooms could help to counteract bone marrow suppression induced by chemo- and radiotherapy (Hofer and Pospisil, 2011). Their main component, ß-glucans, have hematopoietic effects and improve bone marrow regeneration *in vitro* (Sorimachi et al., 2001).

Several reviewed studies showed a possible improved therapeutic response to chemo- and radiotherapy. Tsai et al. observed an advantage in the 6-months OS and mortality rate in favor of *Antrodia cinnamomea* (Tsai et al., 2016). The medicinal mushroom preparation was given for 4 weeks, most likely too short a duration to produce significant changes. *In vitro* studies with *Antrodia cinnamomea* showed inhibition of the growth of breast cancer cells, including MCF-7 cells and tamoxifen-resistant MCF-7 cell lines, and sensitization of radio/chemotherapy of cancer stem cells (CSC) by modulation of microRNA expression (Su et al., 2017; Lin et al., 2018). Another *in vitro* study showed an association between the CSC-inhibitory effect of *Antrodia cinnamomea* and significant downregulation of several microRNAs and cancer stemness expression levels in brain and breast CSCs (Su et al., 2017).

A Cochrane review of five RCTs using *Ganoderma lucidum* showed that cancer patients (especially lung cancer, colon cancer, breast cancer) in the verum group were more likely to respond positively compared to placebo and chemo/radiotherapy alone (RR 1.50; 95% CI 0.90 to 2.51, *p* = 0.02) (Jin et al., 2016). The treatment with *Ganoderma lucidum* alone did not show the same regression rate as the combined therapy.

A meta-analysis including 1,094 patients with curatively resected colorectal cancer treated with polysaccharide K, a main ingredient of *Coriolus versicolor*, showed an OS risk ratio of 0.71 (95% CI: 0.55–0.90; *p* = 0.006). The disease-free survival risk ratio was 0.72 (95% CI: 0.58–0.90; *p* = 0.003) (Sakamoto et al., 2006).

Another meta-analysis including 3,117 patients of 38 RCTs in China showed an overall response rate in treating lung cancer treated with lentinanIV (1–1.5 mg/d, 2–8 weeks, depending on the study), a main ingredient of *Lentinula edodes*, was increased from 43.3% of chemotherapy alone to 56.9% of lentinan plus chemotherapy. Compared with chemotherapy alone, lentinan plus chemotherapy showed more efficacy in treating lung cancer (pooled RR 0.79, 95% CI: 0.74–0.85) (Zhang et al., 2018).

Altogether, adverse effects were rarely reported. Gastrointestinal symptoms were the most common problem, which may be due to the high amount of dietary fiber and prebiotics known to cause mild stomach upset such as bloating, abdominal pain, diarrhea and constipation. Tsai et al. observed a significant decrease in platelet count in patients treated with *Antrodia cinnamomea*, despite the short treatment duration of 30 days (Tsai et al., 2016). The data indicated a large difference in the decline in platelet levels between the two groups in patients with lung cancer (84 vs. 5%) and gastric cancer (22 vs. 5%) which could not be explained properly. Also patients with colorectal cancer treated for 6 months with *Agaricus sylvaticus* showed reduced platelet counts compared to placebo (Fortes et al., 2009). The relevance of a reduced platelet count was unclear and needs further investigation. The assessment of adverse effects of medicinal mushrooms as well as the correct dosage and possible drug interactions must be further clarified in future CTs.

The postulated mechanisms of action are still vague and diverse. Secondary metabolites of medicinal mushrooms may produce health benefits and anticancer effects by activating stress resistance mechanisms, including autophagy, DNA repair and expression of antioxidant enzymes (Kalaras et al., 2017; Martel et al., 2020). Medicinal mushrooms are recognized as immunomodulators and anti-cancer agents that are able to disrupt certain cellular signal transmission pathways, which are associated with cancer development and progression (De Silva et al., 2012). The biochemical mechanisms that mediate this biological activity are still not clearly understood. Several pathways, antiproliferative and immunomodulatory effects triggered by medicinal mushrooms have been described (Aras et al., 2018; Ayeka, 2018; Joseph et al., 2018; Rossi et al., 2018). Mushroom polysaccharides are known to stimulate latent natural killer cells, T cells, B cells and macrophage-dependent immune system responses (Wasser, 2017). Several species of medicinal mushrooms have shown anti-tumour properties in experimental studies (Wasser, 2011). However, much of the evidence is based on results of *in vitro* assays as well as *in vivo* animal data (De Silva et al., 2012).

Compounds of mushrooms may produce hermetic dose-response effects, with potentially beneficial effects at low doses and toxic effects at high doses in normal cells, while in cancer cells only the toxic effects at high doses are potentially beneficial (Calabrese, 2005). The supposed hormetic effects of fungochemicals seem to be relatively modest in humans, which may explain why such effects are observable in animal models but less obvious in clinical studies (where several variables and opposing factors may dilute such effects).

Natural killer cells play an important role in innate immunity by recognizing major histocompatibility class I-negative target cells, which can escape immune surveillance by cytotoxic T cells. A Cochrane review of five RCTs using *Ganoderma lucidum* as complementary cancer therapy showed that patients in the verum group had increased natural killer cell activity and increased levels of CD3, CD4 and CD8, leukocytes, and CD4/CD8 ratio (Jin et al., 2016).

Moreover there is evidence that orally taken medicinal mushrooms have an influence on the microbiome (Friedman, 2016; Jayachandran et al., 2017; Grant et al., 2019). Medicinal mushrooms could act as prebiotics that can stimulate the growth of beneficial microbiota. Prebiotics can modulate the human intestinal microbiota and can attenuate various disease such as diabetes, obesity and cancer (Rossi et al., 2018). The important sources of prebiotics in mushrooms are indigestible polysaccharides, which can inhibit the proliferation of pathogens by promoting the growth of probiotic bacteria in the gut (Singdevsachan et al., 2016). Poor intestinal microbiota can contribute to the development of cancer and various metabolic disorders leading to inflammation in the intestine, liver and brain (Boulangé et al., 2016; Vivarelli et al., 2019). Isolated substances from medicinal mushrooms such as crestin and lentinan administered IV are most likely to have different mechanisms of action *in vivo* than orally taken full-spectrum mushroom formulations. Future studies are necessary to compare the effects of extracts and isolated substances.There is a lack of conclusive evidence on dosage of medicinal mushrooms. First *in vivo* dose escalation studies were identified. Torkelson et al. conducted a controlled dose escalation study with nine breast cancer patients undergoing radiation therapy receiving *Coriolus versicolor* for 6 weeks (Torkelson et al., 2012). A faster recovery was observed in the medium and high dose patients (6 and 9 g/day). Further research is needed to determine the correct dose of medicinal mushrooms, as well as possible interactions with chemotherapeutic agents, in order to make it a safe treatment during conventional cancer therapy. In addition, the quality of the products is of crucial importance, as contamination with mold, pesticides and heavy metals could be a problem for herbal products from natural sources (Harris et al., 2011).

There are several relevant limitations to the reviewed studies, particularly the small study populations (ranging from n = 15 to n = 198), short treatment periods (ranging from 3 weeks to 6 months), studies examining various tumor entities (breast, ovarian, endometrial, colorectal, lung, hepatocellular cancer, multiple myeloma), lack of information about the exact ethnicity of the study participants and non-standardized mushroom preparations. The methodological quality of most studies was unsatisfactory and most results were poorly reported in many respects (especially between-group comparisons were missing quite often). Future studies should be reported with the assistance of the Consolidated Standards of Reporting Trials statement. In addition, detailed information about production, composition and bio-active ingredients were mentioned only in a few of the reviewed studies and there only partially. Furthermore, this review included only English publications. It is likely that further studies can be found in Chinese and Japanese databases. Four out of five RCTs of the Cochrane review were in Chinese and were published in Chinese journals (Jin et al., 2016).

## Conclusion

This narrative review shows possible potential of medicinal mushrooms in complementary cancer treatment. Promising anticarcinogenic effects have been documented *in vitro* and *in vivo* for several medicinal mushrooms. However, only few clinical studies defined OS or time to disease progression as primary endpoints. Others were too short in duration as to be fitting to strengthen this hypothesis. Immunomodulating effects such as the proliferation of lymphatic cell lines, immunoglobulins and cytokines have also been documented in humans.

It is likely that medicinal mushrooms could improve quality of life during and after conventional cancer therapy. Their prebiotic effects pose a possible explanation, as do other, yet unknown effects. A better emotional and physical status, better sleep and less fatigue, as well as fewer side effects of conventional chemotherapy such as nausea, vomiting and gastrointestinal symptoms could be observed in the reviewed CTs on patients taking medicinal mushrooms.

Adverse events of treatment with medicinal mushrooms were poorly reported but appeared to be rare. The relevance of a reduced platelet count remains unclear and requires further investigation. The correct dosage and possible drug interactions also need to be further clarified in future clinical studies. There is an urgent need to investigate efficacy and safety of medicinal mushrooms in well planned CTs as more and more patients use mushrooms as a co-medication.

However, current knowledge does not support the routine use of medicinal mushrooms in cancer patients. The decision whether to use medicinal mushrooms as an add-on treatment in cancer care should remain patients’ preferences for now, assisted by evidence-informed physicians. Currently, evidence for the use of medicinal mushrooms in cancer is rather scarce and the methodological quality of most of the reviewed studies is poor. The existing evidence only allows for preliminary conclusions. The lack of standardization in several aspects of the included studies, such as inconsistent preparation methods and different modes of administration of medicinal mushrooms as well as the lack of key information in the reviewed publications, reduces the reliability and validity of those studies.

A special feature of medicinal mushrooms might be, as they produce hundreds of active compounds, that they may influence several cancer-related processes in a synergistic way. Therefore, not only studies on certain mushroom-derived compounds are warranted, but also further research on complex anticancer effects facilitated by combinations of molecules could be of great interest.

In summary, despite the promising preliminary data, more scientific work needs to be done to clarify the use of medicinal mushrooms in cancer therapy. Particularly, further clinical research is needed, including methodologically high-quality studies, larger sample sizes, standard mushroom preparations and longer-term follow-up studies. In addition, future studies should also investigate the preventive aspects of medicinal mushrooms, as medicinal mushrooms or more common types of mushrooms can also be effective in reducing cancer incidence as part of a healthy diet and lifestyle (Li et al., 2014).

## Author Contributions

MJ conceived the manuscript, gathered information, assembled Table 1, and wrote part of the paper. AM supervised the conception, and wrote part of the paper. DF gathered information, wrote part of the paper, and assembled Table 1. MH, MF, DL and VM wrote part of the paper. CF wrote part of the paper, and integrated the information.

## Funding

This work was funded by the Wilhelm Doerenkamp-Stiftung, Chur, Switzerland. The funder had no role in the design and conduct of the study; collection, management, analysis, and interpretation of the data; preparation, review, or approval of the manuscript; or decision to submit the manuscript for publication. We acknowledge support from the German Research Foundation (DFG) and the Open Access Publication Fund of Charité ‐ Universitätsmedizin Berlin.

## Conflict of Interest

The authors declare that the research was conducted in the absence of any commercial or financial relationships that could be construed as a potential conflict of interest.
